# A Robust Bioassay of the Human Bradykinin B_2_ Receptor That Extends Molecular and Cellular Studies: The Isolated Umbilical Vein

**DOI:** 10.3390/ph14030177

**Published:** 2021-02-24

**Authors:** François Marceau, Hélène Bachelard

**Affiliations:** 1 Département de Microbiologie-Infectiologie et Immunologie, Faculté de Médecine, Université Laval, Québec, QC G1V 0A6, Canada; francois.marceau@crchudequebec.ulaval.ca; 2 Axe Endocrinologie et Néphrologie, Centre de Recherche du CHU de Québec-Université Laval, Québec, QC G1V 4G2, Canada

**Keywords:** bradykinin, B_2_ receptor, kallikrein-kinin system, human umbilical vein

## Abstract

Bradykinin (BK) has various physiological and pathological roles. Medicinal chemistry efforts targeted toward the widely expressed BK B_2_ receptor (B_2_R), a G-protein-coupled receptor, were primarily aimed at developing antagonists. The only B_2_R antagonist in clinical use is the peptide icatibant, approved to abort attacks of hereditary angioedema. However, the anti-inflammatory applications of B_2_R antagonists are potentially wider. Furthermore, the B_2_R antagonists notoriously exhibit species-specific pharmacological profiles. Classical smooth muscle contractility assays are exploited over a time scale of several hours and support determining potency, competitiveness, residual agonist activity, specificity, and reversibility of pharmacological agents. The contractility assay based on the isolated human umbilical vein, expressing B_2_R at physiological density, was introduced when investigating the first non-peptide B_2_R antagonist (WIN 64338). Small ligand molecules characterized using the assay include the exquisitely potent competitive antagonist, Pharvaris Compound 3 or the partial agonist Fujisawa Compound 47a. The umbilical vein assay is also useful to verify pharmacologic properties of special peptide B_2_R ligands, such as the carboxypeptidase-activated latent agonists and fluorescent probes. Furthermore, the proposed agonist effect of tissue kallikrein on the B_2_R has been disproved using the vein. This assay stands in between cellular and molecular pharmacology and in vivo studies.

## 1. The Kallikrein-Kinin System

The kallikrein-kinin system (KKS; defined in Abbreviations) is an endogenous multiprotein cascade whose activation regulate a plethora of physiological processes, including inflammation, coagulation, pain, cell proliferation, blood pressure, vasodilation, smooth muscle contraction, cardioprotection, and vascular permeability, through the subsequent release of vasoactive kinins [1,2,3,4,5,6,7,8]. Kinins are small blood-derived locally acting peptides generated by the proteolytic action of serine proteases, called kallikreins, on circulating precursors, the kininogens, in response to a variety of physiological and pathological stimuli, including ischemia and tissue injury [1,7,9,10]. There are two different precursors of kinins, high- and low-molecular-weight kininogens (HK and LK) and both are encoded by alternative splicing of a single gene (*KNG1*) and produced by the liver. Two types of kallikreins have been identified: tissue kallikrein (KLK-1 protein, *KLK1* gene product), produced as a zymogen in the kidney, salivary glands, vascular endothelial cells, lungs and other tissues [3], and plasma kallikrein, also found as the circulating zymogen prekallikrein (*KLKB1* gene product) [1]. Its proteolytic activation is mediated by the Hageman factor (factor XII, FXII) on negatively charged surfaces (such as the denuded basal membrane of damaged endothelium). Plasma kallikrein preferentially processes HK into bradykinin (BK, a nonapeptide), whereas LK is preferentially cleaved by KLK-1, releasing the decapeptide Lys-BK (or kallidin) [1,2,5,7,11]. Once generated, kinins exert their biological effects through the activation of two distinct G-protein-coupled receptors (GPCRs) termed B_2_ and B_1_ receptors (B_2_R, B_1_R) [7]. The B_2_R subtype shows high affinity for BK and Lys-BK, while the B_1_R subtype is rather responsive to des-Arg^9^-BK and Lys-des-Arg^9^-BK, two fragments of the native kinins, BK and Lys-BK, in which the Arg^9^ residue has been enzymatically removed [7]. These two peptides are the only biologically active metabolites of BK and Lys-BK, respectively. Kinins have strong permeability-enhancing and vasodilatory capacity that need to be tightly controlled to prevent excessive edema.

The B_2_R is constitutively expressed on most cell types, including endothelial cells, some epithelia, sensory neurons, and other cell types [7,12] and accounts for most of the vascular and metabolic actions of BK [6,13,14,15,16]. The most immediate vascular effects of kinin are vasodilation, mediated by the endothelial production of nitric oxide and prostanoids via calcium signaling, and increased vascular permeability and fluid leakage due to a contraction of the endothelial cells [7,17]. These effects are particularly relevant to angioedema states, such as hereditary angioedema (HAE), a rare genetic disorder with unpredictable, self-limiting and localized swelling episodes involving the cutaneous and subcutaneous tissues. The B_2_R undergoes rapid desensitization and internalization after agonist stimulation and receptor phosphorylation [7]. In contrast, the B_1_R have limited distribution and are generally absent in healthy tissues, but may be strongly induced within few hours after noxious stimuli or inflammatory cytokines, such as interleukin (IL)-1β and tumor necrosis factor (TNF)-α [7,18,19]. The induction of B_1_R has been associated with the production of inflammatory mediators, stimulation and recruitment of inflammatory cells, and the activation of several intracellular signaling pathways. The agonist-activated B_1_R is not phosphorylated and relatively resistant to desensitization and internalization, as opposed to the B_2_R [7]. This receptor is thus potentially important in chronic inflammation.

## 2. Hereditary Angioedema as the Therapeutic Showcase of the KKS

Kallikreins are endogenously controlled by circulating serine protease inhibitors (serpins). Among them, the C1 esterase inhibitor (C1-INH; *SERPING1* gene product) is the most important physiological inhibitor of plasma (but not tissue) kallikrein, factor XIa, factor XIIa, and several complement serine proteases [20,21,22,23]. Tissue kallikrein is inhibited by endogenous kallistatin (*SERPINA4* gene product) [24]. C1-INH is a key negative regulatory protein of the proteolytic cascade systems of plasma, the complement, contact system, and intrinsic coagulation. A lack or dysfunction in the C1-INH in blood is causally associated with attacks that involve the excessive stimulation of the endothelial B_2_R, leading to increased microvascular permeability and the formation of subcutaneous and/or submucosal edema, potentially life-threatening if it occurs in the larynx [25,26]. This clinical condition is seen in patients with HAE, is a rare group of autosomal dominant disorders caused by variants of several genes. The most common HAE forms are caused by genetically determined low C1-INH levels in plasma (type I HAE) or a defect in C1-INH activity (type II HAE) [27]. Less common forms of HAE with normal C1-INH are associated with mutation of genes encoding the coagulation FXII (*F12* gene) [28], plasminogen (*PLG*) [29,30] or of kininogens (*KNG1*) [31]. Overall, all these gene variants are proven or postulated to be permissive for kinin production [27].

As it became clear that BK was the primary mediator of angioedema symptoms in HAE, and that a dysregulation of BK pathways was responsible for angiodema attack onset, considerable efforts have been devoted to drug development targeted to components of the kallikrein-kinin system. Thus, among the drugs that have reached clinical use or at least clinical trials to abort or prevent attacks of HAE, a first therapeutic option is to replace the missing protein, by infusing plasma-derived C1-INH (Berinert^®^, Cinryze^®^, and Haegarda^®^), or recombinant C1-INH (Ruconest^®^) [32]. Inhibiting plasma kallikrein is an alternative option. Ecallantide (Kalbitor^®^) is a potent and specific inhibitor of plasma kallikrein. It is a recombinant 60-amino acid long protein that has been engineered from an existing serpin, the human tissue factor pathway inhibitor, and synthesized in the yeast *Pichia pastoris*. Subcutaneously injected ecallantide was proven to be effective to abort attacks of HAE in two clinical trials [33]. Among other plasma kallikrein inhibitors, the recombinant, fully human IgG1, monoclonal antibody lanadelumab (Takhzyro^®^), was recently approved as an orphan drug for prophylaxis in HAE attack prevention. It is a potent and specific inhibitor of plasma kallikrein. The mean elimination half-life of the drug is about two weeks [32,34]. Orally bioavailable, small molecule inhibitors of plasma kallikrein are also being developed, the most advanced being berotralstat [35]. Garadacimab (CSL 312) is a monoclonal antibody that neutralizes factor XIIa, a potentially useful intervention point in HAE because this enzyme activates prekallikrein; it is being evaluated in clinical trials [32,34]. A fourth type of therapeutic approach for HAE is the pharmacologic blockade of B_2_Rs. Icatibant (Hoe 140, Firazyr^®^, Table 1) is the only B_2_R antagonist that has been registered for clinical use in many countries and only for one indication: HAE. When given subcutaneously, the peptide icatibant aborts or limits attacks of HAE of type I and type II and also attacks in patients with normal C1 inhibitor (HAE-nC1 INH) [32,34]. An orally bioavailable class of B_2_R antagonists is currently developed [36] (see below) and the bioassay based on the human umbilical vein has a particular relevance in this context.

**Table 1:** Selected antagonist ligands of the bradykinin B2 receptor (B_2_R) evaluated using the human umbilical vein contractility assay.

Antagonist	pA_2_ ± s.e.m.	Slope ± s.e.m.	Specificity (sp): Inactive against Listed Agonists; Other Remarks	Refs.
*selected peptide antagonists*				
icatibant (Hoe 140)	8.2 ± 0.26	−0.83 ± 0.26	= d-Arg[Hyp^3^, Thi^5^, D-Tic^7^, Oic^8^]-BK; sole B_2_R ligand in clinical use	[37]
	8.00 ± 0.11	−1.06 ± 0.14	sp: 5-HT, histamine	[38]
	8.42 ± 0.07	−0.99 ± 0.06	sp: weak at B_1_R (pA_2_ 5.48)	[39]
	8.18 ± 0.28	≈−1		[40]
	8.06 ± 0.37	−0.85 ± 0.16	sp: B_1_R agonist	[36]
MEN 11270	8.14 ± 0.22	−0.95 ± 0.11	= conformationally constrained derivative of icatibant; sp: 5-HT, noradrenaline	[41]
B-9430	7.70	−1.10	= d-Arg-[Hyp^3^, Igl^5^, D-Igl^7^, Oic^8^]-BK	[42]
B-10380	6.83 ± 0.04	−1.00	B-9430 N-terminally extended with the green-emitting fluorophore carboxyfluorescein-ε-aminocaproyl	[43]
B-10665	6.83		B-9430 N-terminally extended with the infrared-emitting fluorophore Cy7	[44]
*selected non-peptide antagonists*				
WIN 64338	5.99 ± 0.08	−1.15 ± 0.08	sp: histamine, f-Met-Leu-Phe, 5-HT, U46619	[37]
	6.06 ±0.12			[40]
bradyzide	≈ 5.42			[45]
Compound 19c	7.53 ± 0.24	−1.14 ± 0.18		[45]
FR 173657	7.80 ± 0.30	≈−1		[40]
	8.22	−1.00		[46]
anatibant (LF16-0687)	9.1 ± 0.2	≈−1		[47]
	8.3		sp: 5-HT	[48]
	8.46 ± 0.10	−1.11 ± 0.07	pA_2_ against the partial agonist activity of Compound 47a	[49]
FR 172357	8.65	−0.99	sp: 5-HT, noradrenaline, endothelin-1, B_1_R agonist	[50]
Pharvaris Compound 3	9.67 ± 0.42	−0.76 ± 0.13	sp: 5-HT, U46619, B_1_R agonist; reversible at 1–10 nM	[36]

## 3. The Need for a Human Bioassay for the B_2_R Antagonists

While the B_2_R from various mammalian species retains high affinity for BK, it was striking that smooth muscle and other pharmacological assays from diverse laboratory animals were disparate when the effects of synthetic antagonists were considered. Supporting this view, the clinically used peptide antagonist icatibant (Hoe 140; D-Arg[Hyp^3^, Thi^5^, D-Tic^7^, Oic^8^]-BK; Table 1) is insurmountable and nearly irreversible at the rabbit B_2_R (rabbit jugular vein contractility assay) and determines a slow endocytosis of rabbit recombinant B_2_Rs [51,52], but is a partial agonist at the sheep B_2_R [53]. Icatibant also depresses the maximal effect of BK in the guinea pig ileum, unlike an alternate BK-related antagonist sequence [54].

Considering the need of drug developers, a bioassay for the human form of the B_2_R is desirable. Altura et al. [55] initially showed the contractile effects of several agonists, including BK, in the isolated umbilical artery and vein, ethically available from postdelivery sampling of umbilical cord. These tissues are devoid of sympathetic nerve terminals [56] and their sensitivities to catecholamines and angiotensin II is relatively low [55]; however, they are highly responsive to contractile mediators that can be found in stagnant blood, perhaps related to the post-partum physiological closure of the umbilical vessels. Thus, serotonin (5-hydroxytryptamine), thromboxane A_2_, prostaglandin F_2α_, histamine, BK, endothelin-1 and some complement-derived peptides, such as C3a and C5a, are all potent contractile agents in the isolated human umbilical artery and/or vein [55,56,57,58,59]. The contractility assay based on human umbilical vessels is obviously relevant to the human form of the B_2_R and has been formally introduced in 1994 for evaluating antagonists of this receptor, the peptide icatibant and the first nonpeptide antagonist WIN 64338 [37].

**Figure 1:**
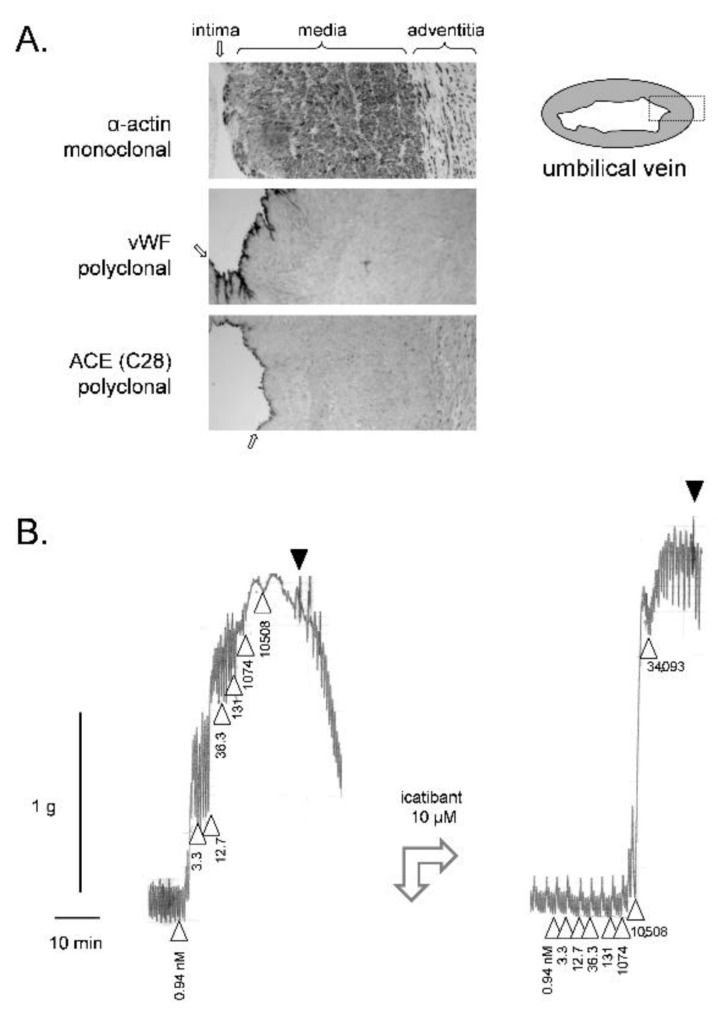
(**A**) Immunohistochemistry for angiotensin-I converting enzyme (ACE) and cell markers (monoclonal anti-α-actin for the smooth muscle cells, polyclonal anti-von Willebrand factor (vWF) for the endothelium) in paraffin sections of the human umbilical vein (100×, approximate width of rectangular fields 700 µm). The dark precipitates indicate positive cells. The position of tunicae intima, media and adventitia are indicated (the intima by arrows). Modified from [63] with permission. (**B**) Left: Construction of a cumulative concentration–effect curve for BK-induced contraction by using a ring of human umbilical vein pre-equilibrated for 3 h. Abscissa scale: time; ordinate scale: isometric contraction, grams. △ indicates the application of BK (cumulative nanomolar concentration indicated) and ▼ the first of a series of washouts. Right tracing recorded in the same tissue at time 5 h: the antagonist icatibant at a high concentration reduced the sensitivity to BK, but not its maximal effect. This experiment was part of a project approved by the local ethics committee (CHU de Québec-Université Laval, File no. 2017-3720). Umbilical cords were obtained following elective caesarean sections. Informed consent was obtained from the mothers.

The microscopic morphology of the human umbilical vein is examined in Figure 1A using markers for smooth muscle cells (α-actin) and vascular endothelial cells (von Willebrand factor and the peptidase angiotensin I converting enzyme (ACE)). The high ratio of smooth muscle cells to endothelial cells possibly explains that no endothelium-mediated vasorelaxation is recorded in this isolated vessel. Indeed, BK-induced contraction is uninfluenced by pharmacological inhibition of endothelial nitric oxide synthase or endothelium abrasion [37]. Furthermore, since ACE mediates the major kinin-clearing metabolic pathway in blood and in vivo [12,60], pharmacologic blockade of ACE does not change the contractile potency of BK [37]. In the contractility assay based on the rabbit jugular vein, a very thin tissue, ACE blockade potentiates BK [61,62], possibly due to a higher endothelium/smooth muscle ratio. Spiral and longitudinal strips of umbilical vessels have been shown to respond similarly to various agents [55]; rings of umbilical vein, well adapted to record contraction, were widely used in the literature covered herein.

The construction of a cumulative concentration-effect curve for BK in the isolated umbilical vein is illustrated in Figure 1B (ring preparation in Krebs’ solution). The threshold concentration is usually 1 nM or slightly below [50]. Despite spontaneous rhythmic activity, the preparation maintains sufficient plateau contractions to record the full curve. One laboratory reported that the addition of the calcium channel inhibitor nifedipine reduced this spontaneous activity without interference with the tonic response to BK [40]. Full relaxation is obtained following repeated washing with fresh buffer. BK potency and maximal effect is stable in a given tissue if the cumulative concentration-effects are constructed at 2 h intervals [37,45]. This allows introducing an antagonist drug in the bathing fluid 30 min before the recording of a second concentration–effect curve. In Figure 1B, a high concentration of icatibant (10 µM) was introduced before constructing the second curve: the apparent potency (EC_50_) was much reduced by this treatment, but not the maximal effect (E_max_) when very high BK concentrations are reached in the bathing fluid, hinting at a competitive (= surmountable) behavior, contrasting with the insurmountable effect of this drug at B_2_R from other mammalian species.

**Figure 2:**
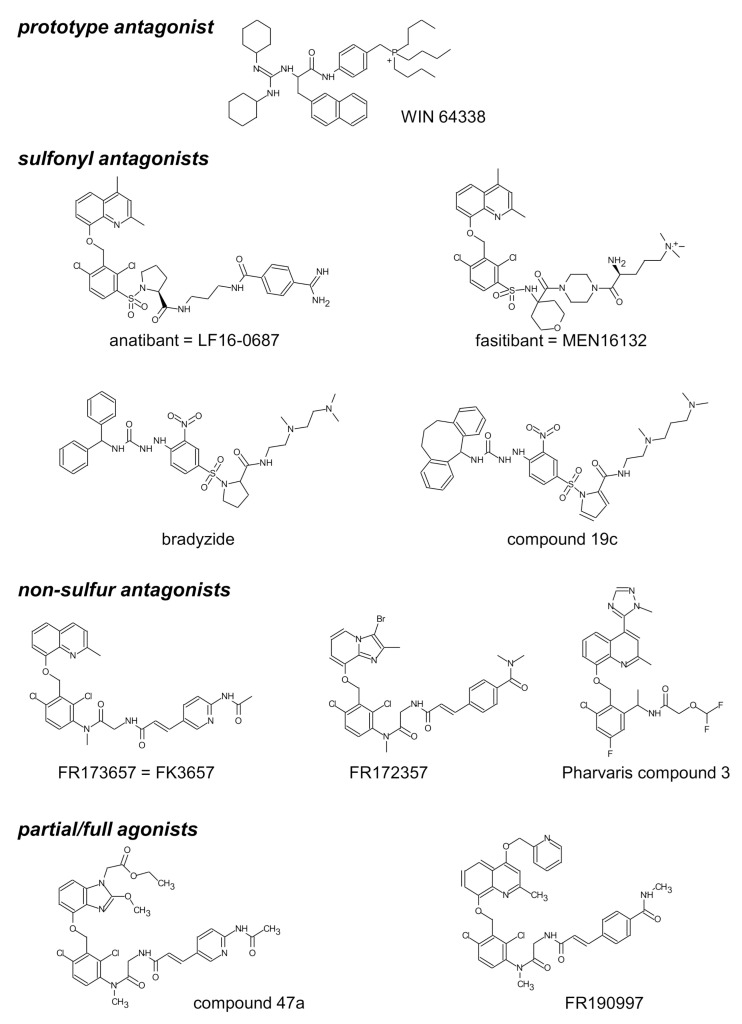
Structure of selected nonpeptide ligands of the B_2_R. Modified from [65] and [36] with permission.

**Figure 3:**
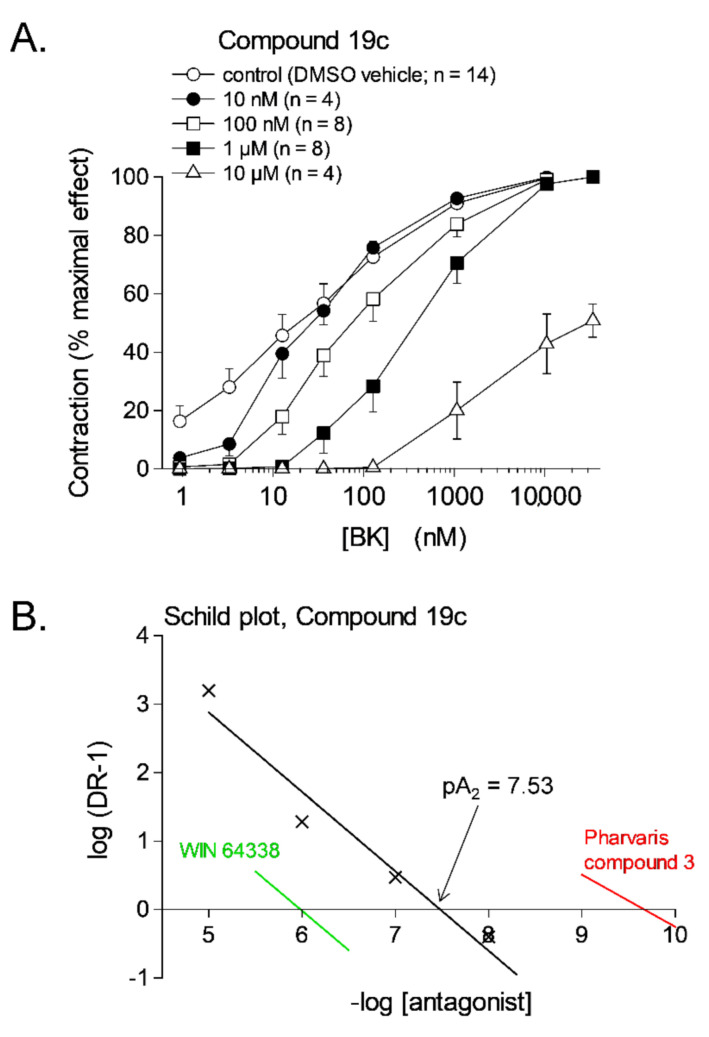
(**A**) Effect of the nonpeptide antagonist of the B_2_R, Compound 19c, on bradykinin (BK)-induced contraction in the human isolated umbilical vein. Each tissue was subjected to the construction of two full cumulative concentration-effect curves, in the absence of antagonist (3 h, not shown) and in the presence of an antagonist or its DMSO vehicle applied 30 min earlier (5 h). Values are the means ± s.e.m. of the number determinations indicated by *n*. For the highest concentration of the antagonist, the maximal effect of BK was evaluated from the separate curve constructed at 3 h. (**B**) Schild plot analysis for Compounds 19c; the x-axis intercepts of the regressions for 2 other nonpeptide antagonists that span the whole potency range (Table 1) are also shown for comparison. Modified from [45] with permission.

Classical pharmacology is recalled analyzing the effect of antagonists in smooth muscle bioassays: well before the era of receptor molecular biology, Schild plot analysis has been developed to quantify the potency of antagonists on the logarithmic pA_2_ scale and also to detect competitive behavior [64]. Thus, tissue treatment with Novartis Compound 19c (Figure 2), one of many nonpeptide antagonists developed since the 1990s, shifts the concentration–effect curve of BK-induced contraction to the right, without depressing E_max_ for antagonist concentrations below 10 µM (Figure 3A); this translates into a pA_2_ value of 7.53, the intercept of the abscissa axis in the regression (Figure 3B), and the competitive behavior is inferred from the regression slope close to −1 (Figure 3B and Table 1).

The structure of selected small molecule antagonists developed since 1994 by various industrial organizations are illustrated in Figure 2. A few that have reached clinical trials have not been introduced in therapeutics (anatibant, fasitibant, possibly FK3657) [36]. Except the low potency phosphonium prototype WIN 64338 [66], significant structural commonalities (“chemotypes”) can be found in this series, and this line of investigation has led to the definition of a minimal pharmacophore for small molecule B_2_R antagonists [67]. All illustrated drugs but one (fasitibant) have been evaluated in the umbilical vein contractility assay by various investigators and those have been found surmountable (Table 1), but with widely different potencies (pA_2_ values). Despite variable technical practices in various laboratories, the potency estimates are usually consistent (Table 1). The species specificity of B_2_R antagonists is illustrated by the pair bradyzide and Compound 19c (Figure 2): while the former drug has a high affinity at the rat B_2_R, is orally bioavailable and active in vivo in the rat, notably as an analgesic [68], it has only a weak affinity for the human form of the receptor (Table 1). A deliberate medicinal chemistry effort, leading to the analog Compound 19c, has optimized its affinity at the human B_2_R [69] and, in parallel, its potency in the umbilical vein assay (Figure 3, Table 1) [45]. Several of the antagonists listed in Table 1 have been found inactive against venous contraction induced by serotonin, histamine, a B_1_R agonist or other agents. The exquisitely potent Pharvaris Compound 3 (Table 1, Figure 2) is surmountable and reversible after washout [36]. This drug is close analog of an orally bioavailable B_2_R antagonist, PHA-022121, active in vivo in a subhuman primate species [70] and currently being clinically developed [71,72]. Pharvaris Compound 3 has a relatively low affinity for the B_2_R from the rat, mouse or dog [36], disqualifying these animal models for that development program. Thus, in time scale of several hours, the umbilical vein assay allows determining potency, competitiveness, specificity and reversibility of B_2_R antagonists.

In addition to interspecies variability, there is another compelling reason to use such a physiological bioassay early when evaluating series of novel B_2_R ligands: the so-called binding paradox. Cost-effective screening assays based on cultured cells, such as the displacement of [^3^H]BK binding or second messenger measurements, provide affinity estimates that are occasionally dissociated with those of more complex bioassays [40]. The ionic strength of binding assay buffers used for radiolabelled BK binding competition is often very low; this may increase the affinity of the ligand-receptor interaction because the protein–protein or protein–peptide affinity is artificially enhanced under these conditions. Thus, a systematic comparison of the inhibition of [^3^H]BK binding to membranes of B_2_R expressing cells in low and normal ionic strength buffers has shown a higher affinity for a series of peptide ligands, including icatibant, under the low strength condition, but no effect for the nonpeptide antagonists WIN 64338 or FR 173657 [40]. The umbilical vein contractility assay, run at physiological Na^+^ concentration, was in general agreement with binding competition data based on normal ionic strength [40]. This was again recently observed when studying the Pharvaris series: icatibant, used as a reference antagonist, was nearly as potent as Compound 3 in the [^3^H]BK binding competition assay run at low ionic strength, but not in the contractility assay where the non-peptide Compound 3 was 41-fold more potent [36].

## 4. Nonconventional Ligands of the B_2_R Assessed Using the Umbilical Vein

While the medicinal chemistry developments related to the B_2_R have been essentially targeted at producing antagonist drugs, interesting agonists have also been produced, apparently in a fortuitous manner. Thus, B-9972 (D-Arg-[Hyp^3^, Igl^5^, Oic^7^, Igl^8^]-BK) is a perfect isomer of the peptide antagonist B-9430 (Table 1), but behaves as a full agonist, notably in the umbilical vein contractility assay [42]. Since both peptides incorporate non-natural amino acid residues, they are potentially resistant to amino- and carboxypeptidases. B-9972 is slightly less potent than BK as a contractile agent in the vein, but, when washed with fresh Kreb’s buffer, tissues maximally contracted with this analog relax more slowly than those washed after a contraction induced by BK [42], suggesting that undetermined peptidases participate to BK clearance in the tissue when the bathing fluid reservoir of the peptide is eliminated. The cardiovascular effects of B-9972 are not potentiated by ACE blockade in anesthetized rats, unlike the effects of BK [73] and, accordingly, B-9972 has only a marginal affinity for ACE as compared to BK in a [^3^H]enalaprilat binding competition assay [63].

Fujisawa scientists developed even more innovative B_2_R agonists by modifying non-peptide antagonists: Compound 47a and FR190997 are examples (Figure 2) [74,75]. In fact, both compounds behave as partial agonists with a high intrinsic activity (nearly full agonists) in the human umbilical vein contractility assay [49,50,76]. Their contractile effects were competitively antagonized by conventional B_2_R antagonists, anatibant for 47a and icatibant for FR190997 [49,50]. It is striking that these nonpeptide stimulants produce very slowly developing contractions; on washing, the relaxation of tissues is even slower than that of B-9972-stimulated veins. They have also a propensity to desensitize the tissue to further stimulation, variable according to the applied protocol [49,50]. This may be related to the persistent endocytosis of the ligand-B_2_R complexes followed by receptor destruction induced by metabolically resistant agonists such as Compound 47a and B-9972 in cellular systems, whereas BK has a reversible effect with complete recycling of the B_2_R population at the cell surface [42,49]. Therapeutic applications of the nonpeptide partial agonists such as FR190997: have been proposed: topically, as agents that decrease excessive intraocular pressure via B_2_R stimulation in intraoccular structures following efficient tissue penetration [77] and in oncology, where B_2_R downregulation following persistent endocytosis may be salutary in tumor cells where overexpressed kinin receptors fuel proliferation [78].

Other unusual kinin receptor ligands are conjugates of agonist or antagonist peptides. The most successful B_2_R bifunctional peptide ligands were the fluorescent ones [65]. Thus, N-terminally extended analogs of the peptide antagonist B-9430 with green- or infrared-emitting fluorophores were evaluated as BK antagonists in the umbilical vein assay, with only slight losses of affinity relative to the parent peptide (Table 1). The agonist versions, carboxyfluorescein-ε-aminocaproyl-BK and Cy7-B-9972, did not fare as well, exhibiting large potency losses relative to their parent peptide, but were nevertheless usable in the micromolar concentration range [44,79]. These tools were essentially adapted to epifluorescence microscopy of cultured cells expressing B_2_R at high densities.

**Figure 4:**
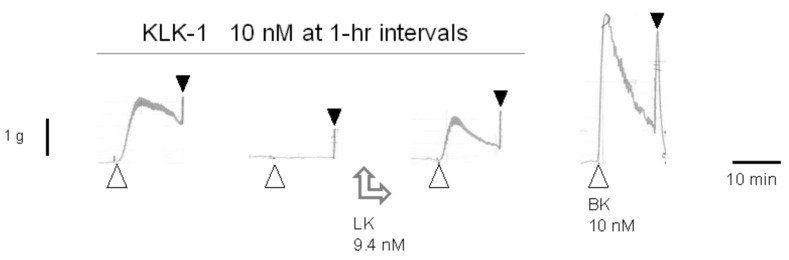
Effect of low-molecular-weight kininogen (LK) replenishment (9.4 nM applied between the second and third stimulation) on tissue kallikrein (KLK-1)-induced contraction in the isolated human umbilical vein. LK restored the effect of the protease in desensitized tissues. Presentation as in Figure 1B. Modified from [81] with permission.

The claim that tissue kallikrein (KLK-1) binds directly to and activates the human B_2_R [80] was disproved in part by using the human umbilical vein assay [81]. A pharmaceutically refined and catalytically active form of KLK-1 contracted the isolated vein via the B_2_R, but in a tachyphylactic manner, without desensitization of the tissue to exogenously added BK. The contractile effect of KLK-1 was abolished by pretreating tissues with icatibant or the protease inhibitor aprotinin and restored in other desensitized tissues by introducing purified LK in the bathing fluid (Figure 4). It was concluded that KLK-1 locally released kinins from a limited reservoir of kininogens, most likely LK, associated with the freshly isolated vascular tissue [81]. Indeed, purified active plasma kallikrein failed to contract the isolated vein via B_2_Rs unless HK was introduced in the bathing fluid [82], probably consistent with the depletion of the contact system in coagulated blood contained in the umbilical vein post partum.

## 5. Prodrug B_2_R Agonists Activated by Vascular Peptidases

Apart from its proinflammatory effects, the protective role of kinins in the circulation under physiological and pathological conditions has attracted a great deal of attention with the advent of ACE inhibitors. The interest in BK was prompted by the discoveries that the kininase II, which degrades BK, is identical to the ACE [83] and by growing body of evidence indicating that part the cardioprotective benefits of ACE inhibition must be accounted for by potentiation of BK [84]. BK is recognized as a regulator of blood pressure, renal and cardiac functions, through its ability to trigger the synthesis and release from vascular endothelial cells of vasorelaxant, anti-hypertrophic and anti-atherosclerotic mediators [8,16,85,86,87,88]. These cardioprotective effects and the release of endothelial mediators, such as nitric oxide, prostacyclin and tissue-type plasminogen activator, are examples of potentially beneficial effects mainly involving kinins and the B_2_Rs of endothelial cells [7,86]. Inspired by a “prodrug” strategy, where a therapeutic B_2_R agonist would be activated only at the level of vascular endothelial cells by resident peptidases, latent B_2_R agonists containing the BK sequence have been produced and evaluated in isolated vascular systems [62,89] and in anesthetized animal [73,90,91]. A first extended sequence of BK to be tested was the 13 amino acids peptide, Met-Lys-BK-Ser-Ser, which is reportedly generated by the neutrophil protease PR3 from HK [92]. The most salient findings were that Met-Lys-BK-Ser-Ser is a contractile agonist of the human umbilical veins ∼30-fold less potent than BK but more potent than expected from the radioligand binding competition assay. Moreover, it was found that the ACE inhibitor enalaprilat, further abates the effect of Met-Lys-BK- Ser-Ser, suggesting that the peptidase activates this peptide by removing the C-terminal dipeptide, Ser-Ser. The resulting peptide, likely Met-Lys-BK, was antagonized by the B_2_R antagonist anatibant in the venous contractility assay [62].

These findings paved the way to subsequent studies aiming to further explore the feasibility of extracting the most desirable cardiovascular effects from a controlled stimulation of endothelial B_2_Rs, by cleavable extended BK homologs, according to a prodrug strategy. These peptidase-activated sequences built around the BK sequence were designed to retain little direct affinity for the B_2_R, but to regenerate BK according to the abundance of resident vascular peptidases (see Table 2) [73,89,91]. In these studies, BK regeneration was estimated using the contractility of the human umbilical vein as model of vascular functions mediated by endogenous B_2_Rs.

**Figure 5:**
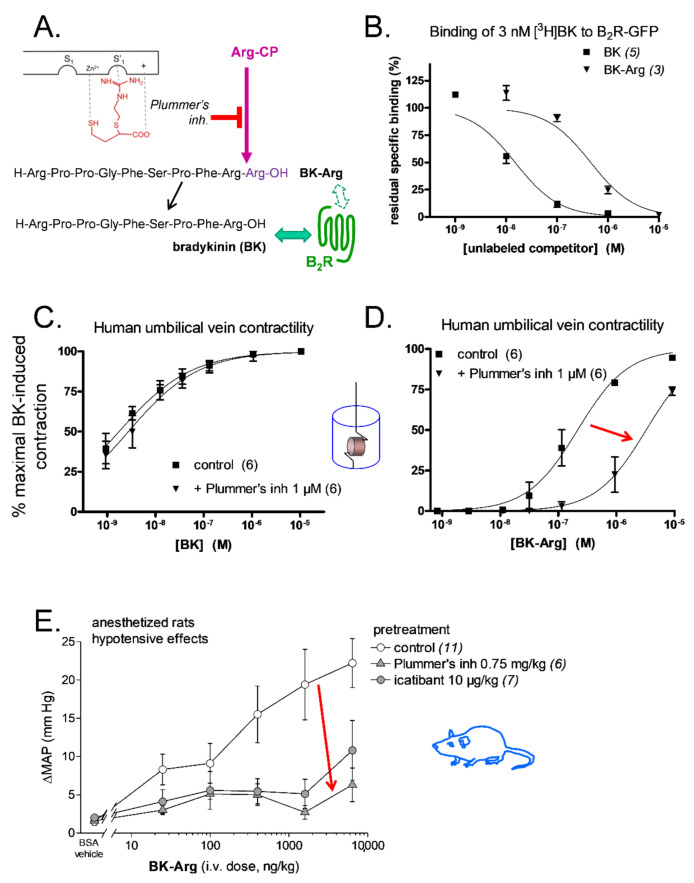
Bradykinin C-terminally prolonged by Arg (BK-Arg), an example of a peptidase-activated latent B_2_R agonist. (**A**) BK-Arg is designed to be activated by widely distributed arginine carboxypeptidases (Arg-CP); Plummer’s inhibitor is an arginine analog that inhibits these peptidases with selectivity. (**B**) BK-Arg has a low affinity for the B_2_R, as evidenced by the competition of the [^3^H]BK binding to a recombinant form of the B_2_R in an assay conducted at 0 °C. (**C**,**D**) Plummer’s inhibitor has no significant effect on the potency of BK in the umbilical vein contractility assay, whereas it reduces that of BK-Arg 15-fold, supporting its metabolism into BK in the tissue. E. In anesthetized rats, the hypotensive effect of BK-Arg (measured as the maximal drop of mean arterial pressure, ΔMAP) is inhibited by pretreatment with either Plummer’s inhibitor or the B_2_R antagonist icatibant. Plummer’s inhibitor had no effect on the hypotensive effect of BK [73] or on the contractile effect of BK (**C**) because Arg-CPs are a minor metabolic pathway for BK inactivation. Red arrows (**D**,**E**) emphasize the effect of Arg-CP blockade. Modified from [73] (**A**,**E**) and [89] (**B**–**D**). Values are means ± s.e.m. of the numbers of replicates indicated between parentheses.

One of the best validated prototypes was BK-Arg, a potential substrate of kininase I-type arginine carboxypeptidases (Arg-CPs) (Figure 5A). BK-Arg was shown to have a 29-fold lower affinity than BK for recombinant B_2_R, as assessed by the binding competition of [^3^H]BK (Figure 5B; Table 2). Interestingly, in the contractility assay, the potency of BK-Arg was found to be significantly reduced (15-fold) in the presence of the Arg-CPs inhibitor, Plummer’s inhibitor, pharmacologically evidencing BK regeneration in situ, while the contractile response to BK was not affected in the presence of this inhibitor. (Figure 5C,D; Table 2) [89]. These results were further supported by pharmacological evidence showing a loss of the hypotensive responses to BK-Arg in anesthetized rats following treatment with the Plummer’s inhibitor or the B_2_R antagonist, icatibant (Table 2 and Figure 5E) [73]. In contrast, the hypotensive responses to BK remained unchanged in the presence of the Plummer’s inhibitor but was inhibited by icatibant (Table 2) [73]. Furthermore, the hypotensive responses elicited by BK-Arg and BK were potentiated in the presence of enalaprilat (Table 2). Considering that BK-Arg has very little direct affinity for ACE, it is thus probable that the enhanced response was consecutive to a potentiation of regenerated BK [73]. Together, these observations support that BK-Arg is an indirect activator of the B_2_R, via its conversion to BK.

Further pharmacological evidence of BK regeneration from extended BK sequences that behave as B_2_R agonists following a limited proteolysis, was based on some other C-terminal extended BK peptides, such as BK-Ser-Tyr, BK-His-Leu and BK-Ala-Pro. These peptides were investigated as potential ACE substrates. As shown in Table 2, when compared to BK these three peptides exhibited an extremely low affinity for recombinant B_2_Rs [89]. Interestingly, BK-Ser-Tyr, BK-His-Leu and BK-Ala-Pro were shown to act as contractile agonists in the vein, that were only 6.9-, 13.5-, and 14.1-fold less potent than BK, respectively, which appears more potent than expected from the radioligand binding competition assay [89]. Moreover, in the presence of enalaprilat, the contractile potency of BK-Ser-Tyr, BK-His-Leu of BK-Ala-Pro decreased 3.8, 19- and 11-fold respectively, suggesting a metabolic activation by ACE of latent B_2_R agonists, especially for BK-His-Leu and BK-Ala-Pro (Table 2) [89]. However, in another study carried out in anesthetized rats, it was found that the intravenous administration of BK-His-Leu or BK-Ala-Pro elicited hypotensive responses, which were abolished by icatibant, but not modified by enalaprilat treatment [73]. Thus, according to these in vivo results, it is likely that when ACE is blocked, the gain of function resulting from BK release from these two C-terminally extended analogs with no affinity for the B_2_Rs, follows a more complex cleavage rule than that anticipated from previous in vitro experiments.

D-Arg-BK-Arg-Arg is another “prodrug” peptide extended around the BK sequence, that was recently tested as a potential B_2_R agonist activated by vascular and blood peptidases. The novel aspect of this construction includes the block of the second kinin inactivation pathway in importance, aminopeptidase P [8,60], by N-terminally extending the BK sequence with D-Arg. This peptide was designed to regenerate D-Arg-BK after two cycles of reaction with Arg-CPs. While D-Arg-BK had a similar affinity to that of BK for rat recombinant B_2_R, the C-terminally prolonged D-Arg-BK-Arg-Arg was found to be 61-fold less potent (Table 2) [91]. Interestingly, in the hUV contractile assay D-Arg-BK-Arg-Arg was found to be a contractile agonist sevenfold less potent than D-Arg-BK, thus again more potent than anticipated from the radioligand binding competition assay [91]. The contractile response to D-Arg-BK-Arg-Arg was found to be only slightly reduced (about twofold) by Plummer’s inhibitor, but more so by enalaprilat (about 3.5-fold), suggesting the removal of the C-terminal dipeptide, Arg-Arg, in a single step by this carboxydipeptidase (Table 2) [91]. In anesthetized rats, both peptides, D-Arg-BK and D-Arg-BK-Arg-Arg were found to be equipotent hypotensive agents following their systemic administration, and these effects were both inhibited by icatibant. Interestingly, in contrast to what was found in the vein contractile assay, enalaprilat treatment had no effect on the hypotensive response to d-Arg-BK-Arg-Arg, while pretreatment with the Plummer’s inhibitor was found to strongly reduce the hypotensive response. Therefore, these in vivo results indicate that Arg-CPs activity is dominant over ACE to regenerate a B_2_R agonist from d-Arg-BK-Arg-Arg, likely d-Arg-BK, following two catalytic steps mediated by Arg-CP. The development of a new prodrug class, designed to stimulate the most desirable effects of endothelial B_2_Rs via the localized generation of a peptide agonist, might find application in intensive care situations where an intravenous line is available.

Another interesting peptide that has been examined for its putative prodrug activity at the human B_2_R is maximakinin (MK), an amphibian 19-mer peptide possessing the C-terminal sequence of BK (Table 2). This peptide was identified as an atypical B_2_R agonist eliciting prolonged signaling [93]. According to radioligand binding competition assays, the affinity of MK at the human recombinant B_2_Rs was found to be much lower than that of BK (~1500-fold), while much smaller reductions relative to BK were seen in rat (~sixfold) or rabbit (~12-fold) recombinant B_2_Rs, substantiating important species-dependent affinity variation for MK across mammalian species [90,93] (Table 2). However, contrasting with the very low affinity of MK for the human B_2_R, MK was shown to be only ~20-fold less potent than BK in the human umbilical vein contractility assay, suggesting the progressive release of shorter and more active peptides following cleavage upstream of the BK sequence. Furthermore, in contrast with the rapid developing contractile response to BK, MK was found to induce a slowly developing contractile response, which was inhibited by icatibant. Interestingly, using liquid chromatography coupled to mass spectrometry (LC-MS), C-terminal fragments, like Lys-Gly-Pro-BK and Gly-Pro-BK were found to be generated from latent MK incubated with human venous tissue, supporting activation via hydrolysis upstream of the BK sequence. Furthermore, the intravenous administration of MK in anesthetized rats was shown to induce a dose-related hypotensive, vasodilator and tachycardic responses, and these effects were all antagonized by pretreatment with icatibant but not enalaprilat, confirming in vivo its resistance to inactivation by ACE (Table 2) [90]. However, the prodrug status of MK in isolated human vascular tissue was evidenced, but without defined activation pathway(s) as various peptidase/protease inhibitors failed to reduce the apparent potency of MK in the umbilical vein (Table 2).

## 6. The B_1_R in the Umbilical Vein

The GPCR most related to the B_2_R is the B_1_R, initially defined as the one mediating the contraction of the isolated rabbit aorta in response to kinins; it was found early that this postulated entity had a peculiar pharmacological profile, the fragment des-Arg^9^-BK, generated by Arg-CPs, being more potent than BK [94]. Furthermore, competitive antagonist peptides such as [Leu^8^]des-Arg^9^-BK, inspired from an early design of angiotensin II receptor antagonists, were developed at the time [94], consolidating the first kinin receptor subtype with both a typical order of potency for agonists and the affinity of a new class of sequence-related antagonist analogs, hence the B_1_R nomenclature [13]. However, many classical smooth muscle preparations responsive to BK (rat uterus, guinea pig ileum, etc.) and in vivo bioassays (hypotensive effect in rats, permeability response in rabbit skin, etc.) did not conform to the B_1_R profile, with extremely weak potency of des-Arg^9^-BK: kinin effects were mediated by a postulated and distinct B_2_R [13]. The consolidation of the B_2_R pharmacological entity awaited the era of molecular biology and the development of the first specific peptide antagonists [7]. It was discovered quite early that the response to des-Arg^9^-BK or Lys-des-Arg^9^-BK in isolated rabbit blood vessels was initially weak or absent but developed as a function of incubation time in vitro in a process dependent on RNA and protein synthesis [95,96], leading to the now widely accepted idea that the gene corresponding to the B_1_R has a strongly regulated expression, to the point of being inducible from a null background [7,97]. Cultured vascular smooth muscle cells or fresh vascular tissue (rabbit and human origin) have been used to establish the role of NF-κB, MAP kinases and Jak/Stat signaling in this process and the accelerating effect of various cytokines such as interleukin-1β, epidermal growth factor and interferon-γ on B_1_R-mediated response intensity [98,99,100,101,102].

The human umbilical vein contractility assay supported investigations of the human B_1_R on two fronts: firstly, the specific pharmacological profile of this entity. Lys-des-Arg^9^-BK (des-Arg^10^-kallidin) is 81-fold more potent than des-Arg^9^-BK as a contractile agent in the vein [39], in line with findings derived from radioligand binding competition exploiting the recombinant human receptor [103]. This potency gap is inferior at the rabbit B_1_R and practically nonexistent at the mouse B_1_R [7], suggesting that the human B_1_R is strongly compartmentalized with KLK-1 that generates Lys-BK (kallidin), mainly from LK, and secondarily Lys-des-Arg^9^-BK. Indeed, in an in vitro reconstitution system, umbilical vein rings in which the B_2_R was blocked with icatibant responded by a B_1_R-mediated contraction if both tissue kallikrein and LK were introduced in the bathing fluid [82]. The previously known B_1_R antagonist Lys-[Leu^8^]des-Arg^9^-BK is competitive and has a high potency against Lys-des-Arg^9^-BK in the venous assay (pA_2_ 7.99) [39]. In another report where the Schild plot analysis has also been applied to the umbilical vein preparation, [Leu^8^]des-Arg^9^-BK pA_2_ was inferior (6.16) [104]. Because peptidase inhibitors, more convincingly if combined, increase the contractile potency of Lys-des-Arg^9^-BK in the umbilical vein, it has been proposed that endogenous ACE, neutral endopeptidase and aminopeptidase N contribute to the inactivation of the optimal B_1_R agonist [105]. The B_1_R agonist Sar-[d-Phe^8^]des-Arg^9^-BK is protected from both amino- and carboxypeptidases and had been known from some time as a long-acting hypotensive agent in LPS-pretreated rabbits [106]. Côté et al. [107] exploited the venous contractility assay and that early design to develop specific B_1_R agonists 80–100 time more potent; for instance, Sar-Lys[Hyp^3^, Igl^5^, d-Phe^8^]des-Arg^9^-BK, with an EC_50_ of 0.62 nM, produced a contraction that was very slowly decreasing on repeated washing of the tissue, not unlike that elicited by inactivation-resistant B_2_R agonists (Section 4).

The other front for which the umbilical vein assay was exploited is the analysis of the B_1_R expression. Thus, the maximal effect of B_1_R agonists is weak at the beginning of the in vitro incubation and progresses as a function of time (typically investigated up to 6 h postmounting) [39,104,108], whereas the responses mediated by B_2_Rs are stable if established at 2-h intervals [37]. In approaches inspired in part from the previous study of the sensitization of the isolated rabbit aorta to des-Arg^9^-BK, the development of the maximal response mediated by the B_1_R in the umbilical vein is depressed by the protein synthesis inhibitor cycloheximide, by dexamethasone and pharmacological inhibitors of NF-κB and MAP kinase signaling [102,109].

## 7. Conclusions

The isolated human umbilical vein is a valuable and ethical bioassay that remains useful to this day due the critical species-dependent pharmacology of the B_2_R antagonists [36]. Valuable features also include a physiological density of receptors, normal ionic strength of the assay buffer and stability over several hours. Sophisticated pharmacodynamic issues like competitiveness, partial agonist activity, reversibility and specificity are readily tested. The evaluation L-alanyl-histamine, a latent agonist of the histamine H_1_ receptors activated by vascular aminopeptidase N [110], illustrate the study of alternate receptor types and peptidases naturally expressed by the umbilical vein. The umbilical vein assay for B_2_Rs has an intermediate level of complexity between cellular and molecular pharmacology on one hand, and in vivo studies in subhuman primates on the other [70].

## Figures and Tables

**Table 2:** Latent agonists of the B_2_R that contain the BK sequence evaluated using the umbilical vein contractility assay ^a^.

Peptide	Loss of Affinity vs. BK, Competition of [^3^H]BK Binding to Recombinant B_2_R	Effect of Peptidase Inhibitor on Apparent Potency, hUV Contractility Assay		In Vivo Validation: Inhibitor Modulation of Hypotensive Effect in Rats	Ref.
		Plummer’s Inhibitor	Enalaprilat		
BK	-	none	none	Plummer’s inh ↔ enalaprilat ↑	[62,73,89]; Figure 5C
Met-Lys-BK-Ser-Ser	>100-fold ↓	NT	12-fold ↓	NT	[62]
BK-Arg	29-fold ↓	15-fold ↓	NT	Plummer’s inh ↓ enalaprilat ↑	[73,89]; Figure 5D
BK-Ser-Tyr	102-fold ↓	NT	3.8-fold ↓	NT	[89]
BK-His-Leu	363-fold ↓	NT	19-fold ↓	enalaprilat ↔	[73,89]
BK-Ala-Pro	490-fold ↓	NT	11-fold ↓	enalaprilat ↑	[73,89]
d-Arg-BK-Arg-Arg	61-fold ↓	2-fold ↓	3.5-fold ↓	Plummer’s inh ↓ enalaprilat ↔	[91]
Maximakinin ^b^	human B_2_R: 1500-fold ↓ rat B_2_R: 6-fold ↓	NT ^c^	NT	enalaprilat ↔	[90]

^a^ Symbols: ↓ decrease; ↑ increase; ↔ no significant effect. NT: not tested. ^b^ Maximakinin is Asp-Leu-Pro-Lys-Ile-Asn-Arg-Lys-Gly-Pro-BK. ^c^ No effect of bestatin + puromycin, EDTA calcium disodium, leupeptin, E-64, omapatrilat or pefabloc SC on maximakinin potency.

## Abbreviations

ACE	angiotensin-I converting enzyme
Arg-CP	arginine carboxypeptidase
B1R	bradykinin B1 receptor
B2R	bradykinin B2 receptor
BK	bradykinin
C1-INH	C1-esterase inhibitor
EC50	half maximal effective concentration
Emax	maximal effect
*F12*	gene encoding factor XII
FXII	coagulation factor XII
GPCR	G protein coupled receptor
HAE	hereditary angioedema
HK	high molecular weight kininogen
IL-1	interleukin-1
KKS	kallikrein-kinin system
KLK-1	tissue kallikrein
*KLK1*	gene encoding tissue kallikrein
*KLKB-1*	gene encoding plasma prekallikrein
*KNG1*	gene encoding kininogens
LC-MS	liquid chromatography–mass spectrometry
LK	low molecular weight kininogen
Lys-BK	lysyl-bradykinin, kallidin
pA2	a measure of affinity of the antagonist for its receptor
*PLG*	gene encoding plasminogen
s.e.m.	standard error of the mean
*SERPINA4*	gene encoding kallistatin
*SERPING1*	gene encoding C1-INH
TNF-α	tumor necrosis factor-α
vWF	Von Willebrand factor
